# Long-term effects of early antibiotic intervention on blood parameters, apparent nutrient digestibility, and fecal microbial fermentation profile in pigs with different dietary protein levels

**DOI:** 10.1186/s40104-017-0192-2

**Published:** 2017-08-01

**Authors:** Miao Yu, Chuanjian Zhang, Yuxiang Yang, Chunlong Mu, Yong Su, Kaifan Yu, Weiyun Zhu

**Affiliations:** Jiangsu Key Laboratory of Gastrointestinal Nutrition and Animal Health, Laboratory of Gastrointestinal Microbiology, College of Animal Science and Technology, Nanjing Agricultural University, Nanjing, Jiangsu 210095 China

**Keywords:** Antibiotics long term effect, Antimicrobial, Blood parameters, Low protein diet, Metabolites, Microbiota

## Abstract

**Backgroud:**

This study aimed to determine the effects of early antibiotic intervention (EAI) on subsequent blood parameters, apparent nutrient digestibility, and fecal fermentation profile in pigs with different dietary crude protein (CP) levels. Eighteen litters of piglets (total 212) were randomly allocated to 2 groups and were fed a creep feed diet with or without in-feed antibiotics (olaquindox, oxytetracycline calcium and kitasamycin) from postnatal d 7 to d 42. On d 42, the piglets within the control or antibiotic group were mixed, respectively, and then further randomly assigned to a normal- (20%, 18%, and 14% CP from d 42 to d 77, d 77 to d 120, and d 120 to d 185, respectively) or a low-CP diet (16%, 14%, and 10% CP from d 42 to d 77, d 77 to d 120, and d 120 to d 185, respectively), generating 4 groups. On d 77 (short-term) and d 185 (long-term), serum and fecal samples were obtained for blood parameters, microbial composition and microbial metabolism analysis.

**Results:**

EAI increased (*P* < 0.05) albumin and glucose concentrations in low-CP diet on d 77, and increased (*P* < 0.05) urea concentration in normal-CP diet. On d 185, EAI increased (*P* < 0.05) globulin concentration in normal-CP diets, but decreased glucose concentration. For nutrient digestibility, EAI increased (*P* < 0.05) digestibility of CP on d 77. For fecal microbiota, the EAI as well as low-CP diet decreased (*P* < 0.05) *E. coli* count on d 77. For fecal metabolites, on d 77, EAI decreased (*P* < 0.05) total amines concentration but increased skatole concentration in low-CP diet. On d 185, the EAI increased (*P* < 0.05) putrescine and total amines concentrations in low-CP diets but reduced (*P* < 0.05) in the normal-CP diets. The low-CP diet decreased the concentrations of these compounds.

**Conclusions:**

Collectively, these results indicate that EAI has short-term effects on the blood parameters and fecal microbial fermentation profile. The effects of EAI varied between CP levels, which was characterized by the significant alteration of glucose and putrescine concentration.

**Electronic supplementary material:**

The online version of this article (doi:10.1186/s40104-017-0192-2) contains supplementary material, which is available to authorized users.

## Background

In intensive swine husbandry systems, piglets commonly receive in-feed antibiotics during early life stage, mainly aiming to prevent outbreaks of respiratory and gastro-intestinal tract (GIT) infections, and promote growth [1]. However, their enormous and uncontrolled use has led to many resistant bacterial strains, causing detrimental effects in both humans and animals. In China, although the use of antibiotics in feed during weaning period of the pig is still a common practice, it has increasingly received safety concerns from consumers and thus the use of antibiotics in feed will be banned in the future. Thus, to better design alternatives to antibiotics for pigs, it is important to understand the post-term effect and the possible mode of action of the antibiotics. The immediate effects of antibiotic intervention on blood parameters [2], apparent nutrient digestibility [3], gut bacterial composition [4, 5] and microbial metabolism [6, 7] have been described, especially in newly weaned pigs. However, much less attention has been paid to antibiotics intervention in suckling and nursing stage and long-term (carry-over) effects on host metabolism and GIT microbiota after withdrawal of antibiotics. In pigs, early life antibiotic exposure affected pancreatic development and glucose metabolism 5 wk after antibiotic withdrawal [8]. A recent study found piglets that received an subcutaneous injection with 0.1 mL tulathromycin (dosage 2.5 mg/kg body weight) on d 4 after birth showed decreased microbial diversity and an altered microbial composition in jejunal digesta on d 176, but had limited effect on d 56 [9]. Using mice models, previous studies also have indicated that low-dose antibiotic exposure in early life had a long-term effect on host metabolism and adiposity [10, 11]. These results suggest that alteration of gut microbes at early life may have long-lasting effect on subsequent host metabolism and gut microbiota. However, it is still unclear whether the early use of antibiotics in suckling and nursing stage have long-term impact on subsequent metabolism and gut microbes in pigs.

In addition to the early use of antibiotics, dietary protein level could also affect the gut health of the animal [12]. In practice, low-protein amino acid-supplementation diets are used to decrease nitrogen excretion and feed costs. Our previous study found that low crude protein (low-CP) diet increased the counts of Bacteroidetes, *Clostridium* cluster IV and *Clostridium* cluster XIVa, decreased *Escherichia coli* counts, and decreased cadaverine, phenol and indole compounds concentrations [13, 14], suggesting a beneficial effect of low-CP diet. Antibiotics could decrease the nutrient utilization by gut microbiota and increase those available for body metabolism [15]. However, when fed low-CP diet, whether early antibiotic intervention affect body metabolism and fecal microbial fermentation profile in pigs remains unclear.

To test the hypothesis that early antibiotic intervention (EAI) (from d 7 to d 42) may have long-lasting effects on subsequent host metabolism and GIT microbes and microbial fermentation in pigs, the present study examined the blood parameters, apparent nutrient digestibility, and major bacterial taxonomic groups and microbial metabolism in feces on d 77 (short-term) and d 185 (long-term) in relation to different CP diets.

## Methods

### Animals and experimental design

A total of 18 litters crossbred (Duroc ×Landrace × Large White) newborn piglets (212 piglets in total) were used in this study. The piglets on d 7 while with the sow were randomly allotted to two groups (*n* = 9 litters) and offered creep feed (Additional file 1: Table S1) either without (control) or with a mixture of antibiotics (50 mg/kg oxytetracycline calcium, 50 mg/kg kitasamycin, and 50 mg/kg olaquindox) (antibiotic). This mixture of antibiotics is commonly used as a growth promoter for piglets in commercial farms in China in order to ensure healthy transition around weaning. Oxytetracycline calcium is a broad spectrum antibiotic with antibacterial activity against gram-negative and gram-positive bacteria, and kitasamycin exhibits activity mainly against gram-positive bacteria, whereas olaquindox exhibits antibacterial activity both gram-negative and gram-positive bacteria. The dosages for the antibiotics used were according to the dose limitation in Regulations of Feeding Drug Additives (announcement No. 168) approved by Ministry of Agriculture, China. On d 23, the sows were removed from the piglets, while the piglets remained in the environmental-controlled nursery with 1.8 × 2.5 m pens fitted with a hard plastic fully slotted floor, and fed the same creep feed until d 42, which is a typical feeding program in a commercial farms in China. On d 42, the piglets within the control or antibiotic group were mixed, respectively, and then randomly allotted to 1 of 2 dietary treatments (Low-crude protein, Low-CP; Normal-crude protein, Normal-CP, respectively) according to their equal average body weight (BW) and gender, respectively, which generated 4 groups (Control-Low CP, Con-LP; Control-normal CP, Con-NP; Antibiotic-Low CP, Ant-LP; Antibiotic-Normal CP, Ant-NP, respectively). There were 5 pens replicates per treatment group and 9 pigs per pen. The numbers of barrows and gilts were not equal on d 42, but spilt as evenly as possible across treatments (23 gilts and 22 barrows, 22 gilts and 23 barrows, 24 gilts and 21 barrows, and 23 gilts and 22 barrows for treatments Con-LP, Con-NP, Ant- LP, and Ant- NP, respectively).

The diets comprised a normal-CP diet (20%, 18%, and 14% CP during d 42 to d 77, d 77 to d 120, and d 120 to d 185, respectively, supplemented with lysine, methionine, threonine, and tyrptophan) and a low-CP diet (16%, 14%, and 10% CP during d 42 to d 77, d 77 to d 120, and d 120 to d 185, respectively, supplemented with four limiting AA mentioned above) (Table 1). The 2 diets were formulated to meet or exceed NRC [16] nutrient recommendations. Creep feed diets were fed as crumble, whereas normal- or low-CP diets were in a pelleted form. After d 42, the pigs were moved to a total confinement house with 2.5 × 3.0 m pens that had partial concrete slatted floors. During the experimental period, the pigs consumed feed ad libitum and feed residues were recorded daily. All pigs had free access to water via a low-pressure nipple drinker. The body weight (BW) of pigs was recorded at the beginning of the experiment and at the end of each period to determine average daily gain (ADG), average daily feed intake (ADFI), and the ratio of gain to feed (G:F).

**Table 1:** Ingredient and nutrient composition of experimental diets (%, as-fed basis)^a^

Age range, d: Ingredient, %	42 to 77		77 to 120		120 to 185	
	LP-16%	NP-20%	LP-14%	NP-18%	LP-10%	NP-14%
Corn	68.00	63.70	68.90	58.00	82.40	71.10
Soybean meal	18.00	21.00	18.80	29.38	8.71	19.13
Wheat bran	−	−	6.00	8.00	2.00	4.00
Whey power	4.30	4.30	−	−	−	−
Fishmeal	3.20	8.00	−	−	−	−
Soybean oil	2.70	0.70	2.56	1.60	2.50	2.00
Dicalcium phosphate	0.85	0.10	0.78	0.65	0.90	0.80
Calcium carbonate	0.50	0.36	0.89	0.89	0.82	0.90
Sodium chloride	0.30	0.30	0.30	0.30	0.30	0.30
Vitamin mixture^b^	0.20	0.20	0.20	0.20	0.20	0.20
Mineral mixture^b^	0.80	0.80	0.80	0.80	0.80	0.80
L-Lysine	0.65	0.33	0.49	0.17	0.78	0.48
DL-Methionine	0.20	0.09	0.10	−	0.20	0.11
L-Threonine	0.24	0.10	0.15	0.01	0.30	0.15
L-Tryptophane	0.06	0.02	0.03	−	0.09	0.03
Calculated content^c^, %						
ME^d^, MJ/kg	14.02	14.02	13.82	13.80	13.81	13.82
SID AA^e^, %						
Lysine	1.23	1.23	0.99	0.98	0.98	0.99
Methionine + Cysteine	0.68	0.68	0.56	0.56	0.60	0.56
Threonine	0.73	0.73	0.59	0.60	0.61	0.60
Trptophane	0.20	0.20	0.17	0.20	0.18	0.17
Analyzed nutrient composition^f^, %						
Crude protein	16.63	20.11	14.03	18.02	10.18	14.06

^a^The pigs of control and antibiotic groups were divided to 1 of 2 treatment groups (normal vs. low CP diet) after d 42. *LP* low protein diet, *NP* normal protein diet

^b^Provided per kilogram of diet: vitamin A, 2.85 million IU; vitamin D_3_, 0.6 million IU; vitamin E, 67.50 IU; vitamin K_3_, 750 mg; vitamin B_1_,750 mg; vitamin B_2_, 1500 mg; vitamin B_6_, 900 mg; vitamin B_12_, 7.5 mg; nicotinic acid, 7500 mg; folic acid, 300 mg; calcium pantothenate, 3750 mg; biotin, 37.5 mg; vitamin B_4_, 100 mg; antioxidants, 15 mg; Cu (CuSO_4_^.^5H_2_O), 60 mg; Fe (FeSO_4_^.^H_2_O), 51.6 mg; Co (CoCl_2_), 50 mg; Mn (MnSO_4_^.^H_2_O), 25 mg; Zn (ZnSO_4_^.^H_2_O), 21.2 mg; Zn (ZnO), 6 mg; I (KI), 3.9 mg; Se (Na_2_SeO_3_), 3.0 mg; Carrier (Sepiolite) 604.3 mg

^c^Calculate values according to NRC [16]

^d^ME = metabolizable energy

^e^SID AA = standardized ileal digestible AA

^f^Analytical results obtained according to AOAC [17]

### Sample collection and preparation

The fecal samples were collected for 3 consecutive days, from d 75 to 77 and 183 to 185 of age, respectively, during the feeding period in the morning by rectal stimulation. Feces from each day were collected at least 3 pigs from each of the pen replicates and were pooled for each replicate to create composite samples. The feces were then frozen at −20 °C until the analysis of microbial metabolites and dominant bacterial community, which was completed within 2 weeks after sample collection.

On d 77 (28.4 ± 0.6 kg) and d 185 (102.0 ± 0.6 kg), when the experimental pigs were fasted for approximately 12 h, one median BW barrow from each pen was selected for blood sampling (*n* = 5). Blood samples (10 mL) were collected from the jugular vein and serum was obtained by centrifuging at 3000×*g* for 10 min at 4 °C. Thereafter, samples were stored at −20 °C until chemical analysis.

### Nutrient composition and apparent digestibility analysis

The dry matter (DM; procedure 930. 15; AOAC, 2007), crude protein (CP; N × 6.25; procedure 990.03; AOAC, 2007), crude fat (procedure 2003. 06; AOAC, 2007), ash (procedure 942.05; AOAC, 2007), contents of experimental diets and freeze-dried feces were analyzed according to the procedure of the Association of Official Analytical Chemists (AOAC) [17]. The acid insoluble ash (AIA) concentrations were analyzed by the method of ISO (method no.5985; ISO, 2003) [18]. Total tract apparent digestibility of nutrients was calculated based on the content of AIA as a marker in feed and feces, as previously described [19].

### Serum biochemical parameters

Serum total protein, albumin, glucose, urea, cholesterol, triglyceride, high density lipoprotein-cholesterol (HDLC), and low density lipoprotein–cholesterol (LDLC) were measured by the enzymatic colorimetric method using an AU2700 autoanalyzer (Olympus, Tokyo, Japan). Globulin concentration of individual serum samples was calculated by subtracting the amount of albumin from the total protein concentration.

### DNA extraction and quantitative real-time PCR

The total bacterial DNA was extracted from fecal samples (0.3 g) using the bead-beating and phenol-chloroform extraction methods [20]. The qPCR assay was performed on StepOnePlus™ Real-Time PCR System (Life Technologies, Califormia, USA) with SYBR Premix Ex Taq dye (Takala, Bio, Ostu, Japan). The sequences of the selected targets and primers are listed in Table 2. The reaction mixture (20 μL) consist of 10.0 μL SYBR Green Supermix (Bio-Rad), 0.4 μL ROX Reference Dye, 2.0 μL of template DNA, 6.8 μL of double distilled water, and 0.4 μL of each primer set. The standard curves of each bacterial group were generated with 10-fold serial dilutions of the 16S rRNA genes amplified from the respective target strains. Quantitative real-time PCR amplification was performed using the following conditions: initial denaturation program (95 °C for 30 s), denaturation program repeated for 40 cycles (95 °C for 5 s, 60 °C for 30 s), followed by the melting curve program (60-95 °C with a heating rate of 0.1 °C/s). The melting curves were checked after amplification to ensure single product amplification of consistent melting temperature. Quantification was performed in triplicate, and the mean Ct value was calculated. The results were expressed as log_10_ 16S ribosomal DNA gene copies/g feces.

**Table 2:** Primers used for quantification in this study

Target	Primer sequence 5′→3′	Reference
Total bacteria	Forward: CGGTGAATACGTTCYCGG Reverse: GGWTACCTTGTTACGACTT	[38]
Firmicutes	Forward: GGAGYATGTGGTTTAATTCGAAGCA	[39]
	Reverse: AGCTGACGACAACCATGCAC	
Bacteroidetes	Forward: GGARCATGTGGTTTAATTCGATGAT	[39]
	Reverse: AGCTGACGACAACCATGCAG	
*Clostridium* IV	Forward: GCACAAGCAGTGGAGT	[40]
	Reverse: CTTCCTCCGTTTTGTCAA	
*Clostridium* XIVa	Forward: CGGTACCTGACTAAGAAGC	[41]
	Reverse: AGTTTYATTCTTGCGAACG	
*E.coli*	Forward: CATGCCGCGTGTATGAAGAA	[42]
	Reverse: CGGGTAACGTCAATGAGCAAA	
*Lactobacillus*	Forward: AGCAGTAGGGAATCTTCCA	[43]
	Reverse: ATTCCACCGCTACACATG	

### Measurement of fecal microbial metabolites

The short-chain fatty acid (SCFA), amines, ammonia, and phenolic and indolic compounds were selected as markers of GIT microbiota metabolism. The concentrations of SCFA were analyzed by gas chromatography as described previously [21], with slight modifications. Briefly, approximately 0.4 g of fecal samples were weighed into a 2-mL centrifuge tube, 1.6 mL of double distilled water was added. The mixture was vortexed for 10 min until the material was homogenized and then centrifuged at 13,000×*g* for 10 min at 4 °C. A portion of 1 mL of the clear supernatant was transferred into a new tube, and then added 0.2 mL 25% (*w*/*v*) metaphosphoric acid. After homogenization, the mixture was frozen at −20 °C and kept overnight to precipitate the proteins. After thawing, a portion of 100 μL internal standard (0.64% (*w*/*v*) crotonic acid solution) was added. The tubes were vortexed for 1 min and then centrifuged at 13,000×*g* for 10 min at 4 °C. The supernatant was filtered through a 0.22-μm syringe filter and then analyzed on an Aglient 7890B system with a flame ionization detector (Agilent Technologies Inc.). The following column conditions were used: nitrogen was used as the carrier gas with a flow rate of (17.68 mL/min); the oven, detector and injector port temperature were 130 °C, 250 °C, 220 °C, respectively. These acids were identified by their specific retention times and the concentrations determined and expressed as umol/g.

Amine concentrations in feces were determined by high-performance liquid chromatography (HPLC) with a method according to Yang et al. [22]. Briefly, 1.5 g of feces were treated with 3 mL of 5% trichloroacetic acid, homogenized for 10 min and then centrifuged at 3600×*g* for 10 min at 4 °C. The supernatant was mixed with an equal volume of n-hexane and vortexed for 5 min, the water phase (0.5 mL) was transferred into a new tube, and then added with 1.5 mL saturated Na_2_CO_3_, 1 mL dansyl chloride, and 1 mL NaOH (2 mol/L). The mixed solution was heated at 60 °C for 45 min, and then added with 100 μL ammonia (2.8%) to stop the reaction. The mixture was kept in the water bath until the acetone was vaporized under nitrogen at 40 °C. Finally, the sample was extracted with 3 mL diethyl ether. The extracts were dried under nitrogen and then re-dissolved in acetonitrile. The mixture filtered through 0.22-μm syringe filter and then analyzed on an Aglient 1220 Infinity LC system with an UV detector (Agilent Technologies Inc.).

The ammonia concentration in feces was analyzed using UV spectrophotometer according to Chaney and Marbach [23]. Phenolic and indolic compounds concentration was determined by HPLC as previously described [24]. Briefly, 0.1 g of fecal sample was treated with 1 mL acetonitrile, homogenized for 10 min and then frozen at −20 °C for 20 min. Finally, the mixture was centrifuged at 3000×*g* for 10 min at 4 °C. The supernatant was filtered through a 0.22-μm syringe filter and analyzed for phenolic and indolic compounds (i.e., *p*-cresol, skatole, indole, and phenol) using HPLC with an UV detector (Agilent Technologies Inc.).

### Statistical analysis

All data were analyzed using 2 × 2 factorial MIXED procedure of SAS for a randomized complete block design (SAS 9.2 Institute, Cary, NC, USA). The model included the fixed effect of antibiotic, protein level, associated two-interactions and the random errors of a pen or an individual pig. The ADG, ADFI, G:F, digestibility of nutrients, and fecal fermentation profile were evaluated using the pen as the experimental unit. Blood parameters were assessed using the individual pig as the experimental unit. Differences were considered significant at *P* ≤ 0.05, and tendency was declared with 0.05 < *P* < 0.10. When a significant interaction among antibiotic and CP was observed, data were further analyzed by using a one-way ANOVA with Duncan’s multiple range tests. Differences at *P* < 0.05 were identified significant.

## Results

### Growth performance and apparent nutrient digestibility

Growth performance of the pigs was shown in Table 3. For ADG, there was an interaction (*P* < 0.05) between EAI and dietary CP treatment from d 77 to d 185 and d 42 to d 185. The EAI increased ADG (*P* < 0.05) from d 42 to d 185 in the low-CP diets but not in the normal-CP diets. Meanwhile, low-CP diet tended to reduce the ADFI (*P* = 0.08) from d 42 to d 185, but G:F at each feeding period did not differ among treatments.

**Table 3:** Effects of early antibiotic intervention on growth performance of pigs with different protein level diets^1^

Item	Low-CP		Normal- CP		SEM	*P-*value^2^		
	Con	Ant	Con	Ant		Ant	CP	Ant × CP
42 d BW, kg	12.23	12.20	12.28	12.25	0.07	0.851	0.743	0.981
ADG, kg/d								
d 42 to 77	0.50	0.52	0.51	0.51	0.02	0.686	0.972	0.786
d 77 to 185	0.78^b^	0.81^ab^	0.84^a^	0.83^a^	0.01	0.361	0.002	0.048
d 42 to 185	0.71^b^	0.74^a^	0.76^a^	0.75^a^	0.01	0.180	0.001	0.019
ADFI, kg/d								
d 42 to 77	0.72	0.72	0.78	0.78	0.02	0.945	0.140	0.993
d 77 to 185	2.24	2.36	2.44	2.54	0.06	0.349	0.119	0.891
d 42 to 185	1.85	1.94	2.02	2.09	0.05	0.368	0.089	0.895
G:F								
d 42 to 77	0.70	0.73	0.67	0.66	0.02	0.880	0.227	0.675
d 77 to 185	0.36	0.35	0.36	0.33	0.01	0.323	0.787	0.655
d 42 to 185	0.44	0.44	0.44	0.42	0.01	0.555	0.414	0.605

^a, b^Means in the same row with different superscripts differ (*P* < 0.05)

^1^The commercial creep feed with or without in-feed antibiotics (50 mg/kg olaquindox, 50 mg/kg oxytetracycline calcium, and 50 mg/kg kitasamycin) was fed to pig from d 7 to d 42. Thereafter, the Con and Ant group were further randomly assigned to provide a normal (20%, 18%, 14% CP from d 42 to d 77, d 77 to d 120, d 120 to d 185, respectively) or low CP diet (16%, 14%, 10% CP from d 42 to d 77, d 77 to d 120, d 120 to d 185, respectively), respectively

^2^The *P* values indicate main effects for antibiotic (Ant), protein level (CP) and their interaction (Ant x CP), respectively

For apparent nutrient digestibility, no interactions between EAI and dietary CP treatment were observed (Table 4). On d 77, there was no difference of OM digestibility among treatments. The EAI significantly increased CP digestibility (*P* < 0.05) and tended to increase (*P* = 0.06) DM digestibility. The low-CP diet reduced CP and crude fat digestibility (*P* < 0.05). On d 185, digestibility of crude fat was decreased (*P* < 0.001) when pigs fed the low-CP diet, but DM, CP, and OM digestibility were not affected by treatment. Overall, these results indicated that EAI had short-term effect on nutrient digestibility.

**Table 4:** Effects of early antibiotic intervention on total tract apparent digestibility of pigs with different protein level diets^a^

Item, %	Low-CP		Normal-CP		SEM	*P-*value^2^		
	Con	Ant	Con	Ant		Ant	CP	Ant × CP
d 77								
DM	75.24	76.73	74.81	80.52	0.98	0.065	0.368	0.263
CP	60.00	64.84	64.54	73.45	1.75	0.032	0.040	0.498
OM	79.86	79.65	78.31	83.32	0.88	0.173	0.538	0.140
Crude fat	44.61	48.44	53.96	56.88	1.34	0.136	<0.001	0.831
d 185								
DM	77.12	72.61	79.24	71.82	1.67	0.103	0.843	0.666
CP	72.46	72.45	75.08	73.35	0.67	0.550	0.240	0.550
OM	82.93	81.67	82.14	80.54	0.51	0.198	0.372	0.867
Crude fat	50.52	48.48	57.72	56.74	1.34	0.320	<0.001	0.720

*DM* dry matter, *CP* crude protein, *OM* organic matter

^a^The commercial creep feed with or without in-feed antibiotics (50 mg/kg olaquindox, 50 mg/kg oxytetracycline calcium, and 50 mg/kg kitasamycin) was fed to pig from d 7 to d 42. Thereafter, the Con and Ant group were further randomly assigned to provide a normal (20%, 18%, 14% CP from d 42 to d 77, d 77 to d 120, d 120 to d 185, respectively) or low CP diet (16%, 14%, 10% CP from d 42 to d 77, d 77 to d 120, d 120 to d 185, respectively), respectively. ^2^The *P* values indicate main effects for antibiotic (Ant), protein level (CP) and their interaction (Ant x CP), respectively

### Serum biochemical measurements

To dissect whether EAI and dietary CP level affected the host metabolism, basic serum biochemistry was analyzed. On d 77, albumin, glucose, and urea concentrations in the serum revealed interactions (*P* < 0.05) between EAI and dietary CP treatments (Table 5). The EAI increased albumin and glucose concentrations in the low-CP diet, but increased urea concentration in the normal-CP diet. Meanwhile, EAI increased (*P* < 0.05) triglyceride concentration irrespective of dietary CP, and tended to increase concentration of total protein (*P* = 0.08). The low-CP diet reduced (*P* < 0.05) concentrations of total protein and LDLC irrespective of EAI. On d 185, EAI also interacted with dietary CP on the concentrations of globulin and glucose in the serum (*P* < 0.05). The EAI decreased glucose concentration in normal-CP diet, but increased globulin concentration. Furthermore, low-CP diet reduced (*P* < 0.01) concentrations of urea, triglyceride, and HDLC irrespective of EAI. In general, these results indicated that EAI had short-term effects on serum parameters, and these effects may be influenced by dietary CP levels.

**Table 5:** Effects of early antibiotic intervention on serum biochemical indexes of pigs with different CP level diets^1^

Item	Low-CP		Normal-CP		SEM	*P-*value^2^		
	Con	Ant	Con	Ant		Ant	CP	Ant × CP
d 77								
Total protein, g/L	62.00	66.21	63.61	70.79	1.67	0.082	0.031	0.643
Albumin, g/L	17.73^b^	23.20^a^	18.04^b^	19.45^b^	0.66	<0.001	0.122	0.049
Globulin, g/L	44.27	43.01	45.57	51.34	1.73	0.553	0.178	0.342
Glucose, mmol/L	4.39^c^	5.58^a^	5.44^ab^	5.26^b^	0.14	0.004	0.189	0.002
Urea, mmol/L	2.12^b^	2.16^b^	2.46^b^	3.26^a^	0.12	0.003	<0.001	0.003
Cholesterol, mmol/L	1.87	1.89	1.97	2.09	0.05	0.890	0.223	0.080
Triglyceride, mmol/L	0.45	0.60	0.63	0.75	0.03	0.039	0.145	0.323
HDLC, mmol/L	0.77	0.81	0.84	0.88	0.02	0.132	0.651	0.241
LDLC, mmol/L	1.03	0.99	1.04	1.14	0.04	0.430	0.045	0.603
d 185								
Total protein, g/L	66.62	65.14	65.26	69.34	1.45	0.378	0.342	0.072
Albumin, g/L	31.60	32.64	33.48	31.78	0.73	0.664	0.489	0.081
Globulin, g/L	35.02^ab^	32.50^b^	31.78^b^	37.56^a^	1.56	0.313	0.567	0.022
Glucose, mmol/L	4.54^b^	4.88^b^	5.54^a^	4.78^b^	0.12	0.245	0.021	0.011
Urea, mmol/L	4.38	4.12	5.02	6.04	0.26	0.352	0.004	0.134
Cholesterol, mmol/L	2.10	1.97	2.15	2.20	0.07	0.793	0.322	0.542
Triglyceride, mmol/L	0.46	0.45	0.61	0.57	0.03	0.684	0.020	0.743
HDLC, mmol/L	0.85	0.77	0.91	0.95	0.02	0.593	0.011	0.151
LDLC, mmol/L	0.99	0.99	1.01	0.91	0.04	0.572	0.712	0.574

*HDLC*, high density lipoprotein-cholesterol, *LDLC*, low density lipoprotein–cholesterol

^a-c^Means in the same row with different superscripts differ (*P* < 0.05)

^1^The commercial creep feed with or without in-feed antibiotics (50 mg/kg olaquindox, 50 mg/kg oxytetracycline calcium, and 50 mg/kg kitasamycin) was fed to pig from d 7 to d 42. Thereafter, the Con and Ant group were further randomly assigned to provide a normal (20%, 18%, 14% CP from d 42 to d 77, d 77 to d 120, d 120 to d 185, respectively) or low CP diet (16%, 14%, 10% CP from d 42 to d 77, d 77 to d 120, d 120 to d 185, respectively), respectively. ^2^The *P* values indicate main effects for antibiotic (Ant), protein level (CP) and their interaction (Ant x CP), respectively

### Fecal bacterial populations

**Fig. 1:**
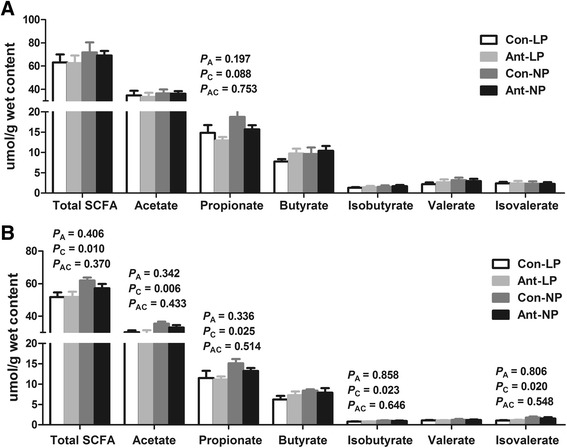
Effects of EAI on SCFAs concentrations in the feces of pigs with different CP levels diets. (Con-LP, ; Ant-LP, ; Con-NP, ; Ant-NP, ). **a** On d 77. **b** On d 185. EAI: early antibiotic intervention. Total SCFA, total short-chain fatty acid. The commercial creep feed with or without in-feed antibiotics (50 mg/kg olaquindox, 50 mg/kg oxytetracycline calcium, and 50 mg/kg kitasamycin) was fed to pig from d 7 to d 42. Thereafter, the control and antibiotic group were further randomly assigned to provide a normal (20%, 18%, 14% CP from d 42 to d 77, d 77 to d 120, d 120 to d 185, respectively) or low CP diet (16%, 14%, 10% CP from d 42 to d 77, d 77 to d 120, d 120 to d 185, respectively), respectively. The *P* values indicate main effects for antibiotic (A), protein level (C) and their interaction (AC), respectively

In order to evaluate the effect of EAI on the quantitative change of bacterial community in the feces, real-time PCR was performed on some major bacterial groups. As shown in Additional file 1: Figure S1, dietary treatments had no effects on fecal major bacterial population on d 77 and d 185, such as total bacteria, Firmicutes, Bacteroidetes, *Clostridium* cluster IV, and *Clostridium* cluster XIVa. However, on d 77 (Fig. 1a), EAI significantly decreased (*P* < 0.05) the counts of *E. coli*, and tended to reduce the counts of *Lactobacillus* (*P* = 0.09). Meanwhile, the low-CP diet also reduced the count of *E. coli* (*P* < 0.05). On d 185 (Fig. 1b), pigs with low-CP diet tended to record a lower count of *Lactobacillus* (*P* = 0.09). Overall, these results suggest that the effect of EAI and dietary CP levels on bacterial groups in the fecal samples was limited except for *E. coli.*

### Fermentation metabolites in fecal contents

To understand whether EAI affect fecal fermentation profiles with different CP diets, fermentation metabolites were determined. For SCFA (Fig. 1), EAI and low-CP diet did not affect fecal total SCFA, acetate, butyrate, isobutyrate, valerate, and isovalerate concentrations on d 77 (Fig. 1a), but the low-CP diet tended to reduce (*P* = 0.088) propionate concentration. On d 185 (Fig. 1b), EAI did not change the SCFA concentrations among groups in the present study. However, the low-CP diet decreased (*P* < 0.05) total SCFA, acetate, propionate, isobutyrate, and isovalerate concentrations.

Ammonia was produced through amino acids deamination by bacteria. On d 77, EAI did not affect the concentration of ammonia (data not shown). The low-CP diet significantly decreased (*P* < 0.05) ammonia concentration in feces. On d 185, dietary treatment did not affect ammonia concentration.

**Fig. 2:**
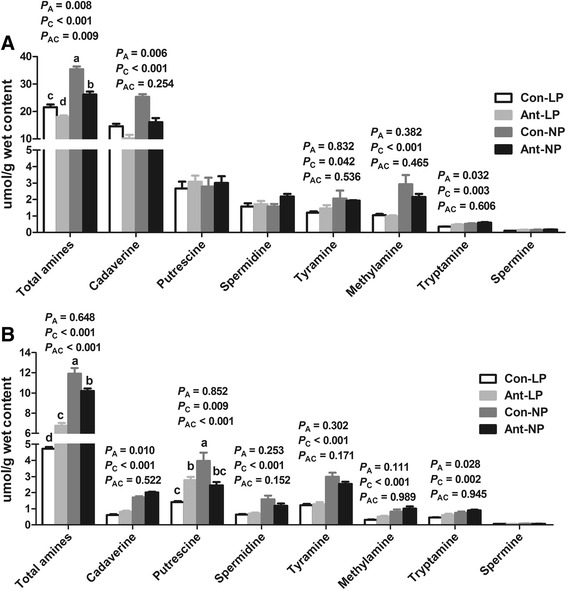
Effects of EAI on amines concentrations in the feces of pigs with different CP levels diets. (Con-LP, ; Ant-LP, ; Con-NP, ; Ant-NP, ). **a** On d 77. **b** On d 185. EAI: early antibiotic intervention. The commercial creep feed with or without in-feed antibiotics (50 mg/kg olaquindox, 50 mg/kg oxytetracycline calcium, and 50 mg/kg kitasamycin) was fed to pig from d 7 to d 42. Thereafter, the control and antibiotic group were further randomly assigned to provide a normal (20%, 18%, 14% CP from d 42 to d 77, d 77 to d 120, d 120 to d 185, respectively) or low CP diet (16, 14, 10% CP from d 42 to d 77, d 77 to d 120, d 120 to d 185, respectively), respectively. The *P* values indicate main effects for antibiotic (A), protein level (C) and their interaction (AC), respectively

Amines are formed from the decarboxylation of proteins, amino acids, and other nitrogenous compounds in the GIT. As shown in Fig. 2, cadaverine and putrescine are the main amines. On d 77 (Fig. 2a), EAI significantly decreased (*P* < 0.05) the concentration of total amines, cadaverine, and increased (*P* < 0.05) the concentration of tryptamine. The low-CP diet significantly decreased (*P* < 0.05) cadaverine, tyramine, methylamine, and tryptamine concentrations. However, the concentrations of putrescine, spermidine, and spermine were not affected by dietary treatment. On d 185 (Fig. 2b), there was an interaction (*P* < 0.001) between EAI and dietary CP level for putrescine. The EAI reduced (*P* < 0.05) concentrations of putrescine in normal-CP diet, but increased its concentration of (*P* < 0.05) in low-CP diet. Additionally, EAI increased (*P* < 0.05) the concentrations of tryptamine and cadaverine. The low-CP diet decreased (*P* < 0.01) the concentrations of total amines, methylamine, tryptamine, putrescine, cadaverine, tyramine, and spermidine.

**Fig. 3:**
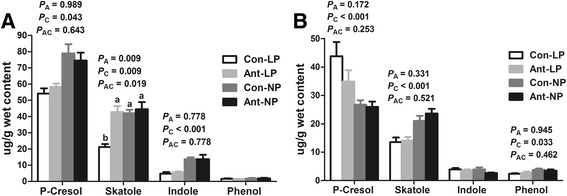
Effects of EAI on phenolic and indole compounds concentrations in the feces of pigs with different CP levels diets. (Con-LP, ; Ant-LP, ; Con-NP, ; Ant-NP, ). **a** On d 77. **b** On d 185. EAI: early antibiotic intervention. The commercial creep feed with or without in-feed antibiotics (50 mg/kg olaquindox, 50 mg/kg oxytetracycline calcium, and 50 mg/kg kitasamycin) was fed to pig from d 7 to d 42. Thereafter, the control and antibiotic group were further randomly assigned to provide a normal (20%, 18%, 14% CP from d 42 to d 77, d 77 to d 120, d 120 to d 185, respectively) or low CP diet (16, 14, 10% CP from d 42 to d 77, d 77 to d 120, d 120 to d 185, respectively), respectively. The *P* values indicate main effects for antibiotic (A), protein level (C) and their interaction (AC), respectively

Phenolic and indolic compounds originate from the intestinal degradation of aromatic amino acids. As shown in Fig. 3, there was an interaction (*P* < 0.05) between EAI and dietary CP level for skatole concentration on d 77 (Fig. 3a). The EAI significantly increased (*P* < 0.05) concentration of skatole in low-CP diets, but not in normal-CP diet. Furthermore, pigs with low-CP diet reduced the concentrations of *p*-cresol and indole (*P* < 0.05). There was no dietary treatment effect on phenol concentration. On d 185 (Fig. 3b), the EAI did not affect the concentrations of phenol, *p*-cresol, and skatole, but tended to reduce (*P* = 0.08) indole concentration. The low-CP diet decreased (*P* < 0.05) the concentration of phenol and skatole but increased (*P* < 0.05) concentration of *p*-cresol. Collectively, these results indicated that EAI had a great impact on metabolites of protein fermentation in feces, but had little effect on carbohydrate fermentation. The low-CP diets reduced the protein fermentation products in feces on both d 77 and d 185.

## Discussion

By employing a whole growth stage continuous feeding strategy, the current experiment enabled to evaluate the short- (77 d) and long-term (185 d) effect of EAI (from d 7 to d 42) on pig blood parameters, apparent nutrient digestibility, microbial metabolism, and bacterial counts in feces with different dietary CP level. Our results suggested that the EAI had short-term effects on blood parameters and microbial fermentation products in pigs, and that these effects were influenced by dietary CP levels.

In the intestine, there are differences in microbiome between gut segments and between fecal samples and intestinal samples. Along the hindgut fermentation in the pig (from cecum to distal colon), proportionally carbohydrate fermentation decreases while proteolytic fermentation increases. However, fecal samples are easy to access and easy for practice, and also can partly, though not exactly, reflect the general microbial metabolism in the gut. Thus, fecal samples are commonly used in many researches for host metabolism in humans and animals. In the present study, we intended to reflect the general gut metabolism through fecal samples analysis and the results showed that an alteration in microbial fermentation profiles of amino acid by dietary treatment, indicating that EAI and CP level affected the gut microbial composition and function. This approach by using fecal analysis can provide a feasible way to study the effects of EAI and CP on fermentation profiles of microbial amino acid metabolism.

### Effects of early antibiotic intervention on growth performance and blood parameters with different protein levels after antibiotic withdrawal

At present, information on the effects of EAI on subsequent growth performance under different dietary CP levels is limited. Skinner et al. [25] reported that the use of antibiotics (therapeutic dose usage) in the nursery diets with normal-CP level appeared to reduce subsequent ADG of the pigs as compared to non-treated controls. In the present study, the antibiotics at low dosage did not have effect on subsequent ADG with normal-CP diet, though increased the subsequent ADG under low-CP diet. Thus, the antibiotic effect on subsequent growth performance may vary depending on the antibiotic dosage and the diet CP level.

The level of serum parameters of animals reflects the body metabolic status according to their internal and external environments [26]. EAI exerted an effect on body metabolism on d 77, such as a significant increase of the concentrations of albumin and glucose, especially in low-CP diets, but not in normal-CP diets. A previous study also indicated that pigs received therapeutic amoxicillin exposure at birth through postnatal d 14 increased glucose concentration at postnatal d 49 [8]. Additionally, we found that the antibiotics effect on serum biochemistry was diminished on d 185. Thus, antibiotic intervention showed short-term effect on body metabolism, but had no long-term effect.

### Effects of early antibiotic intervention on fermentation profiles of microbial amino acid metabolism with different protein levels after antibiotic withdrawal

Antibiotics are usually considered the most cost-effective way to reduce pathogenic bacteria [1]. Based on the microbiota analyses, we found that EAI had limited effect on the bacterial composition, only decreased the numbers of *E. coli* counts in the fecal samples irrespective of dietary CP levels on d 77, which suggested that EAI had limited long-term impact on fecal microbiota. Janczyk et al. [27] observed that piglets treated with amoxicillin (15 mg/kg body weight) by intramuscular administration at d 1 showed a decreased microbial diversity in colon on d 39. Collectively, these findings suggest that EAI less affected bacterial communities in feces.

EAI also affected profiles of biogenic amimes in the pig feces. The significant decrease of cadaverine and total amines concentrations suggested a decreased amino acid decarboxylation after EAI treatment. High concentration of cadaverine can exert detrimental effects on the host, such as inducing DNA damage and oxidative stress [28]. Thus, the decrease of cadaverine and total amines concentration by EAI may exert a beneficial influence on the host. Coincidently, among the bacteria examined in the present study, *E. coli* species are a major protein-fermenting group [29]. Our previous study indicated that the cadaverine concentration was positively correlated with the abundance of *E. coli* [12]*.* Therefore, the decrease of cadaverine concentration by EAI may be due to the decrease in protein-fermenting bacteria counts (*E. coli*) and the subsequent reduction of the catabolism of precursor amino acid (lysine). EAI increased putrescine concentration in low-CP diet but decreased it in normal-CP diet. This results indicates that the effect of EAI on subsequent concentrations of amines may vary with CP level. Furthermore, we found that cadaverine concentration varied between d 77 and d 185. Cadaverine is derived from decarboxylation of lysine [30]. In the current study, the CP level and lysine concentration in the diet was much higher on d 77 than 185 d, which may lead to the variation in cadaverine concentration across ages.

On the other hand, EAI also changed aromatic amino acid metabolism by the microbes in the GIT. Tryptophan, one of the aromatic amino acid, can be decarboxylated into tryptamine by GIT microbes [30, 31]. In the present study, EAI increased tryptamine concentration in the feces irrespective dietary CP levels on d 77 and d 185, suggesting that EAI had a continuous effect on aromatic amino acid metabolism. Tryptamine can stimulate the secretion of serotonin by enterochromaffin cells, and then regulate intestinal motility [32].Thus, an increase of tryptamine may affect the function of intestine. Additionally, EAI also increased skatole concentration on d 77, especially in low-CP diets. Skatole is produced from tryptophan catabolism by GIT bacteria [30]. The increase of skatole concentration indicates that EAI also affected tryptophan catabolism in the gut. Collectively, combined with serum parameters and microbial fermentation profiles, the effects of EAI persisted to short-term after withdrawal, but weakened at finishing period of the pigs.

### Low-CP diets affected host metabolism and fermentation profiles of microbial amino acid metabolism

In our study, low-CP dietary treatment affected the urea metabolism as reflected by the decreased urea concentration in serum both on d 77 and d 185, which has also been found in other reports [33, 34]. Urea is the main nitrogenous end product of amino acids catabolism in pigs. A decrease in serum urea level is indicative of a more efficient use of dietary nitrogen [35]. Thus, our study may indicate that low-CP diet affected nitrogen metabolism in pigs.

Corresponding to the decrease in urea concentration in serum, low-CP diet markedly affected the microbial metabolism of amino acids in the intestine, as shown by the decrease in concentration of most amines, suggesting decreased microbial decarboxylation. Furthermore, low-CP diet decreased some phenolic and indole compounds concentrations. Indole and skatole are products of tryptophan metabolism originating from dietary and endogenous protein [31]. Although the concentration of tryptophan in the normal-CP diet is equal to that of the low-CP diet in the current study, crystalline amino acid is quickly absorbed before reaching the hindgut, and then reduced the availability of substrates for microbial fermentation in the hindgut. Overall, these findings suggest that low-CP diets significantly reduced the microbial fermentation of protein or amino acids and the concentration of potentially harmful metabolites derived from microbial metabolism of feed-derived amino acids. Thus, the dietary nitrogen was used more efficiently with low level of potentially harmful metabolites in pigs fed low-CP amino acids-supplemented diets.

Low-CP diets altered the markers of microbial carbohydrate metabolism on d 185, such as decreased the concentrations of acetate, propionate, isobutyrate, isovalerate, and total SCFA, consistent with Le et al. [36], who observed that reducing dietary CP level from 15% to 12% led to a decrease in total SCFA, acetate, and propionate concentrations for finishing pigs. The concentration of SCFA produced mainly depends on the amount and composition of substrate and the type of microbes present in the GIT [37]. Thus, in the present study, a decrease of SCFAs may be related with the altered metabolism activity of gut microbiota after low-CP diet.

## Conclusions

In conclusion, combined with the changes in blood metabolites, digestibility, and microbial metabolite, our results showed that EAI (from d 7 to d 42) showed short-term effects on pigs, especially on the body and microbial metabolism. The effects of EAI varied between CP levels, which was characterized by the significant alteration of glucose and putrescine concentration. The low-CP diet significantly decreased the amino acids fermentation products in feces.

## Additional file

Additional file 1: Table S1. Ingredients and chemical composition of creep feed diets. **Figure S1.** Effects of early antibiotic intervention on bacterial abundance in the feces of pigs with different CP levels diets. (Con-LP, *White*; Ant-LP, *Light gray*; Con-NP, *Dark gray*; Ant-NP, *Black* ). A: On d 77. B: On d 185. The commercial creep feed with or without in-feed antibiotics (50 mg/kg olaquindox, 50 mg/kg oxytetracycline calcium, and 50 mg/kg kitasamycin) was fed to pig from d 7 to d 42. Thereafter, the control and antibiotic group were further randomly assigned to provide a normal (20%, 18%, 14% CP from d 42 to d 77, d 77 to d 120, d 120 to d 185, respectively) or low CP diet (16%, 14%, 10% CP from d 42 to d 77, d 77 to d 120, d 120 to d 185, respectively), respectively. The *P* values indicate main effects for antibiotic (A), protein level (C) and their interaction (AC), respectively. (DOC 576 kb)

## Abbreviations

ADFI: average daily feed intake
ADG: average daily gain
AIA: acid-insoluble ash
BCFA: branched-chain fatty acid
BW: body weight
CP: crude protein
DW: dry matter
EAI: early antibiotic intervention
G:F: the ratio of gain to feed
GIT: gastrointestinal bacteria
HDLC: high density lipoprotein cholesterol
LDLC: low density lipoprotein cholesterol
OM: organic matter
SCFA: short chain fatty acid
