# Supplementary material for: Long-term effects of early antibiotic intervention on blood parameters, apparent nutrient digestibility, and fecal microbial fermentation profile in pigs with different dietary protein levels
Source: J Anim Sci Biotechnol. 2017 Aug 1;8:60. doi: 10.1186/s40104-017-0192-2 (PMC5537924; doi:10.1186/s40104-017-0192-2)

**Supplementary Tables and Figures**

**Long-term effects of early antibiotic intervention on blood parameters, apparent nutrient digestibility, and fecal microbial fermentation profile in pigs with different dietary protein levels**

Miao Yu, Chuanjian Zhang, Yuxiang Yang, Chunlong Mu, Yong Su, Kaifan Yu and Weiyun Zhu^†^

Jiangsu Key Laboratory of Gastrointestinal Nutrition and Animal Health, Laboratory of Gastrointestinal Microbiology, College of Animal Science and Technology, Nanjing Agricultural University, Nanjing, Jiangsu 210095, China

^†^Corresponding author: Weiyun Zhu. E-mail: [zhuweiyun@njau.edu.cn](mailto:zhuweiyun@njau.edu.cn)

Address: College of Animal Science and Technology, Nanjing Agricultural University, Nanjing, Jiangsu 210095, China

**Additional file 1: Table S1**

**Additional file 1: Table S1.** Ingredient and nutrient composition of creep feed (%, as-fed basis)^1^

| Ingredients (%) | |
| --- | --- |
| Corn | 40.00 |
| Rice, broken | 15.00 |
| Soybean meal, fermented | 10.00 |
| Soybean meal, de-hulled | 6.00 |
| Spray dried animal plasma | 5.00 |
| Whey powder | 7.00 |
| Fish meal | 4.00 |
| Sugar | 4.50 |
| Glucose | 3.00 |
| Soybean oil | 1.50 |
| L-Lysine-HCl (98%) | 0.30 |
| L-Methionine | 0.15 |
| L-Threonine | 0.20 |
| L-Tryptophan | 0.05 |
| L-Isoleucine | 0.05 |
| L-Valine | 0.05 |
| Sodium chloride | 0.30 |
| Limestone | 1.10 |
| CaHPO4 | 0.80 |
| Vitamin mixture^1^ | 0.20 |
| Mineral mixture^2^ | 0.80 |
| Total | 100.00 |
| Nutrient composition, % |  |
| Crude protein | 20.20 |
| Digestible energy (Mcal/kg) | 3.40 |
| Total calcium | 0.85 |
| Total phosphorus | 0.70 |
| Digestible Lys | 1.45 |
| Digestible Met+Cys | 0.79 |
| Digestible Thr | 0.81 |
| Digestible Trp | 0.23 |
| Digestible Ile | 0.74 |
| Digestible Leu | 1.45 |
| Digestible Val | 0.89 |
| Analyzed nutrient composition, % |  |
| Crude protein | 20.13 |

^1^ Vitamin mixture supplied the following per kg complete diet: vitamin A, 15,000 IU; vitamin D3, 3,000 IU; vitamin E, 150 mg; vitamin K3, 3 mg; vitamin B1, 3 mg; vitamin B2, 6 mg; vitamin B6, 5 mg; vitamin B12, 0.03 mg; niacin, 45 mg; vitamin C, 250 mg; calcium pantothenate, 9 mg; folic acid, 1 mg; biotin, 0.3 mg; choline chloride, 500 mg.

Mineral mixture supplied the following per kg complete diet: Fe, 170 mg; Cu, 150 mg; I, 0.90 mg; Se,0.2 mg; Zn, 150 mg; Mg, 68 mg; Mn, 80 mg; Co, 0.3 mg.

**Additional file 1: Figure S1.**

Effects of EAI on bacterial abundance in the feces of pigs with different CP levels diets.

(Con-LP, v
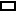
; Ant-LP,
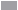
; Con-NP,
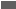
; Ant-NP,
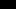
). A: On d 77. B: On d 185.

EAI: early antibiotic intervention. The commercial creep feed with or without in-feed antibiotics (50 mg/kg olaquindox, 50 mg/kg oxytetracycline calcium, and 50 mg/kg kitasamycin) was fed to pig from d 7 to d 42. Thereafter, the control and antibiotic group were further randomly assigned to provide a normal (20%, 18%, 14% CP from d 42 to d 77, d 77 to d 120, d 120 to d 185, respectively) or low CP diet (16%, 14%, 10% CP from d 42 to d 77, d 77 to d 120, d 120 to d 185, respectively), respectively. The *P* values indicate main effects for antibiotic (A), protein level (C) and their interaction (AC), respectively.


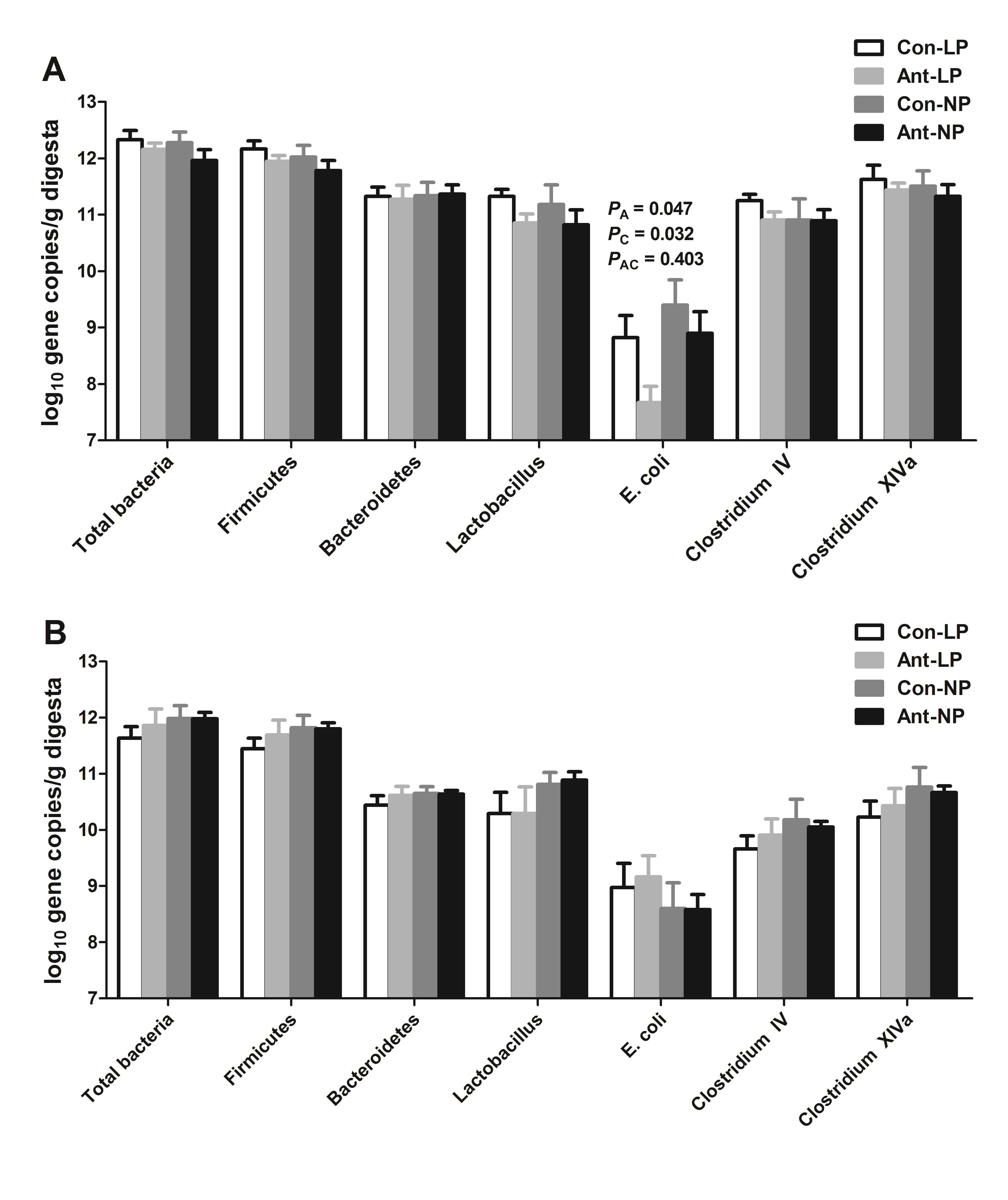

Supplement: Additional file 1: Table S1. — Ingredients and chemical composition of creep feed diets. Figure S1. Effects of early antibiotic intervention on bacterial abundance in the feces of pigs with different CP levels diets. (Con-LP, White; Ant-LP, Light gray; Con-NP, Dark gray; Ant-NP, Black ). A: On d 77. B: On d 185. The commercial creep feed with or without in-feed antibiotics (50 mg/kg olaquindox, 50 mg/kg oxytetracycline calcium, and 50 mg/kg kitasamycin) was fed to pig from d 7 to d 42. Thereafter, the control and antibiotic group were further randomly assigned to provide a normal (20%, 18%, 14% CP from d 42 to d 77, d 77 to d 120, d 120 to d 185, respectively) or low CP diet (16%, 14%, 10% CP from d 42 to d 77, d 77 to d 120, d 120 to d 185, respectively), respectively. The P values indicate main effects for antibiotic (A), protein level (C) and their interaction (AC), respectively. (DOC 576 kb) [file 40104_2017_192_MOESM1_ESM.doc]
